# Why do people choose courts to resolve disputes? A fuzzy-set analysis of Chinese citizens’ judicial reliance

**DOI:** 10.3389/fpsyg.2022.1015987

**Published:** 2023-01-05

**Authors:** Xiang Wang, Changwei Guo, Yuwen Lyu, Shouchao Zhu

**Affiliations:** ^1^Zhou Enlai School of Government, Nankai University, Tianjin, China; ^2^Business School, Renmin University of China, Beijing, China; ^3^School of Marxism, Guangzhou Medical University, Guangzhou, China; ^4^Law School, Dalian Maritime University, Dalian, China

**Keywords:** judicial reliance, judicial professionalism, China, fuzzy-set qualitative comparative analysis (fsQCA), court

## Abstract

We use the concept of judicial reliance to describe the willingness and extent to which citizens seek the help of the court in a dispute. There are obvious local differences in the degree of judicial reliance in different provinces, with some citizens more willing to resort to the courts to settle disputes, whereas others are indifferent to the courts. Based on the judicial survey data of 31 provinces in China, we use fuzzy-set qualitative comparative analysis to explore the possible reasons for differences in citizens’ judicial reliance in China. We find that citizens’ judicial reliance is deeply influenced and restricted by five judicial conditions: transparency, corruption, independence, procedure, and professionalism. These causal conditions influence and interact with each other, thus forming six configurations that produce high judicial reliance. Among these six configurations, judicial professionalism is always the core condition. In recent years, China has indeed attached great importance to the construction of judicial professionalism, which not only optimizes the quality of the internal personnel of the court but also strengthens the public’s recognition of the court.

## Introduction

In the classical theory of judicial politics, judicial power arises from a special practical need—to resolve disputes. Martin Shapiro made a classic argument in this regard, arguing that the triad for purposes of conflict resolution is the basic social logic of courts. When there is a dispute between two people, the disputing parties tend to seek a neutral third party to decide right and wrong and apply the rules of judgment agreed by both parties, thus forming the triad of conflict resolution. But the triad is highly volatile, not only dependent on the consent of the disputing parties but also in danger of turning into a two-on-one relationship. With the emergence and development of the modern state, the state established court institutions to manage this jurisdiction, and formal institutions gradually replaced consent and enforced the application of uniform rules of law (Shapiro, 2013). In Shapiro’s judicial theory system, the court is an intermediary organization specialized in resolving disputes, and the legitimacy of the court’s judgment comes from the “consent” of both parties. In China, civil litigation and administrative litigation mainly depend on the initiation of the parties. The law also gives the parties the right to initiate some criminal proceedings, such as minor criminal cases and criminal cases that should be investigated by the public prosecution organ but are not investigated. This means that the parties can either choose the court to resolve the dispute or resort to other means to solve the problem. So, what determines people’s preference for dispute resolution? What’s the underlying logic? This is the main concern of this article.

In the context of China, the executive power (the government) generally occupies a dominant position, while the judicial power (the court) has a relatively low position of power, because the judicial organs have relied heavily on the local government for human, property and other resources for a long time, so the judicial independence is relatively poor (Jiang, 1998; Ma and Nie, 1998; Fan, 2013; Zhang, 2014), resulting in the problem of “local judicial protectionism.”^1^ Influenced by complex external factors, local courts are relatively independent, judges often pervert the law, and judicial corruption is relatively serious (Li, 2012; Wang, 2015; Ng and He, 2017). The internal system of the court organization is also relatively weak, and the degree of litigation justice and procedural guarantee is insufficient. Judges’ professionalism level is low, and their comprehensive quality is insufficient (Jin and Yan, 2011; Ye, 2015; Tian, 2018). Such long-standing problems have formed negative stereotypes for people, and the court seems to be a less sacred and neutral image in people’s minds (Xu and Chen, 2014). In particular, the media often report negative news about some courts, which deepens people’s distrust (Long, 2013). As a result, when people encounter disputes, they often do not first think of going to court to solve the disputes but will use other means to solve the disputes, such as petition, mediation, arbitration, or other noninstitutional means, such as collective action or violence (Jinhua, 2010). A recent empirical study conducted a large-scale social survey in China, in which 38.14% of nearly 2000 respondents preferred mediation to resolve disputes, and people would also consider other methods: “Seek help from fellow villagers’ associations” (3.56%), “Seek help from people’s organizations such as trade unions and women’s federations” (4.58%), “apply for administrative adjudication or reconsideration” (5.59%), “seek help from other social organizations” (8.47%), “directly go to leaders to report problems (including higher-level leaders)” (9.66%), “solve problems by violence” (1.02%), “gathering people to block the government, blocking roads and other means to make things big to the government Pressure (0.34%) and complaints to the news media (3.39%), while only 18.13% chose the courts to resolve disputes (Zhu and Yuan, 2019).

Xi Jinping once pointed out that “*there are some problems in judicial activities, such as judicial injustice, unjust, false and wrong cases, judicial corruption, money cases, power cases, and human cases. If these problems are not addressed quickly, they will seriously affect the progress of comprehensively advancing the rule of law and social equity and justice*.” That is why the fourth Plenary Session of the 18th Communist Party of China (CPC) Central Committee was dedicated to studying and making decisions on major issues concerning comprehensively advancing the rule of law. The 19th National Congress of the CPC set out new tasks for advancing the rule of law in all respects, defining that by 2035, the rule of law should be established in the country, government, and society. China’s judicial reform has been going on for more than 40 years, and many measures addressed these issues directly, trying to restore the image of the courts and improve their standing. So, have judicial reforms in recent years changed people’s stereotypes about the court? When people encounter disputes, will they choose the court to settle the dispute? And what factors affect and restrict people’s choices? This is the main issue of this article.

**Figure 1:**
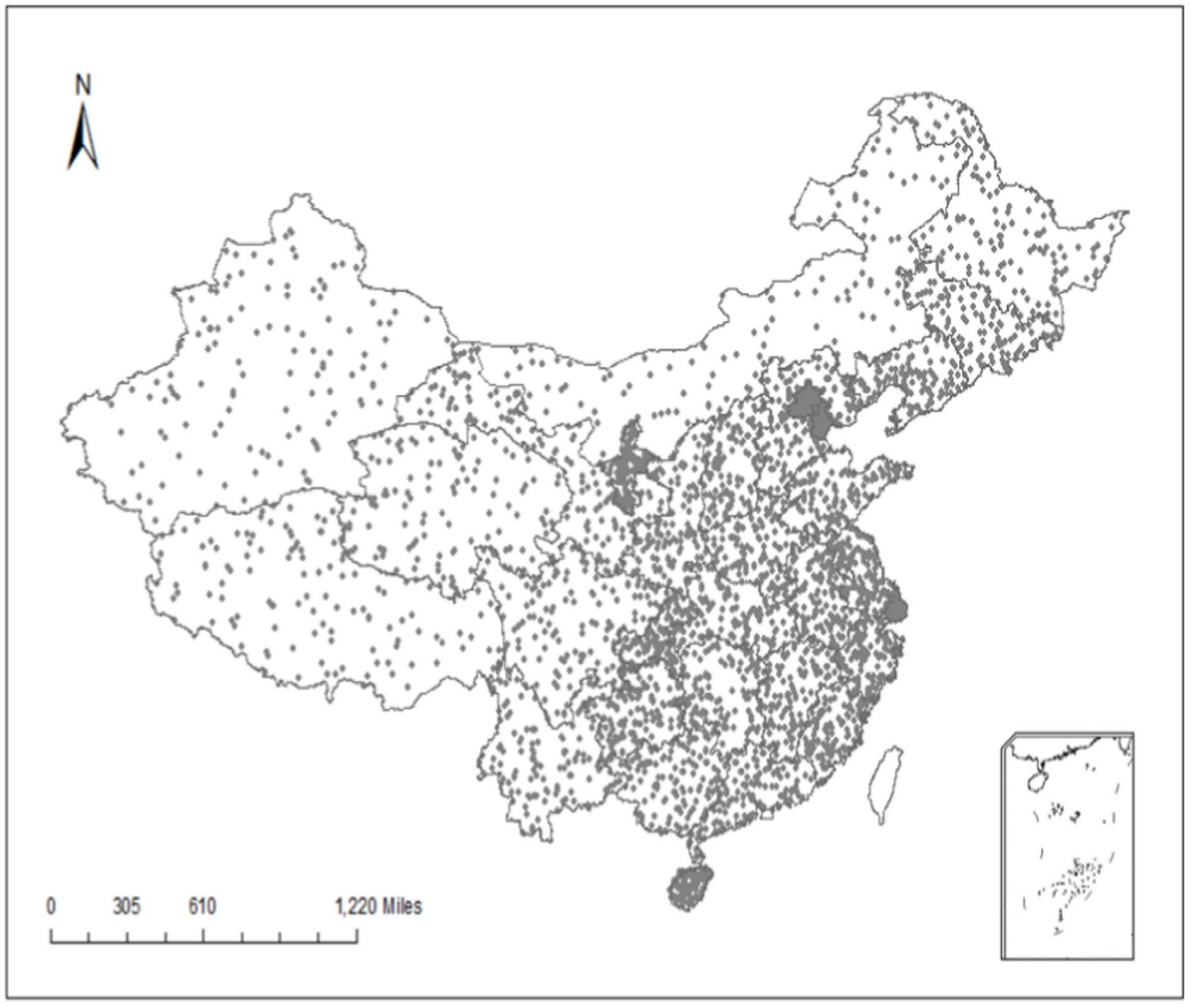
The geographical distribution of judicial reliance in China.

The object of this article is the judicial reliance of Chinese citizens. We use the concept of “judicial reliance” to describe the dependence of citizens on the court when they encounter disputes, mainly reflecting the willingness of citizens to seek the help of the court when they encounter disputes. The Report on Chinese Justice Index 2019 (PCJI) released by the Collaborative Innovation Center of Chinese Judicial Civilization in 2019, in particular, takes a survey of our concern with judicial reliance (“In your area, how likely is it for the parties to take the initiative to go to court to resolve a dispute?”), Figure 1 shows the judicial reliance of different Chinese provinces in 2019, with higher scores indicating more willingness of citizens to resort to justice. There are significant local differences in the judicial reliance of citizens in different provinces. Some provinces have very high judicial reliance (Beijing, Jiangsu, Ningxia, Heilongjiang, etc.), whereas others have very low judicial reliance (Hainan, Qinghai, Shanghai, etc.). So, what accounts for these local differences? What’s the underlying logic? To address this concern, this article integrates two sets of data resources: one is the PCJI, which has conducted a questionnaire survey on the judicial situation of 31 provinces in China, including legal professionals and the public; Second, we have first-hand field research data in many provinces. Based on previous research, we identify five causal conditions that may affect judicial reliance—judicial transparency, corruption, independence, procedure, and professionalism—and construct a corresponding theoretical framework.

Through fuzzy-set qualitative comparative analysis (fsQCA), we find that the existence of any of these five causal conditions alone is not sufficient to lead to high judicial reliance, so the interactive effects of these five conditions should be further analyzed from a configurational view. The different permutation and combinations of these five causal conditions produce six configurations that may bring high judicial reliance. It is worth noting that there is the same core condition in these six configurations: judicial professionalism. Even if there is judicial corruption occupying the core condition, judicial professionalism can effectively offset the negative effects brought by judicial corruption, resulting in high judicial reliance in a region. This also explains, to some extent, why China’s judicial reform in recent years has always put the reform of judicial professionalism in a prominent position. The significance of judicial professionalism is not only to optimize the personnel structure inside the court but also to bring good social effects. Judicial professionalism strengthens citizens’ recognition of the court. Due to their trust in the court, citizens will rely more on the court (rather than other noninstitutional means) to solve problems when they encounter disputes. Our conclusion is also relatively optimistic: With the progress of judicial professionalism, more citizens will rely more on the institutionalized channels provided by courts to resolve disputes, and China’s social stability mechanism will also gain strength.

In the rest of this article, we first construct the theoretical analysis framework based on the previous literature. Then, we introduce the research data and method of this article, according to the characteristics of the data we collect, mainly using fuzzy-set analysis. Section “Data and analytic approach” presents the causal conditions and outcome of the study and introduces how these conditions are measured and calibrated to the fuzzy set. Section “Results” mainly shows the results of our analysis. We identify six configurations that may affect judicial reliance. Section “Discussion: China and judicial professionalism” summarizes our findings and discusses the significance of judicial professionalism to judicial reliance and the reform of the judicial system.

## Theoretic framework: What conditions affect citizens’ judicial reliance

When citizens have disputes, we all know that there are a variety of options available, such as conciliation, mediation, arbitration, and so on. Submitting a lawsuit request to the court, on the other hand, is an important institutional remedy. So, what determines people’s preference for dispute resolution? As a result, we use a notion called “judicial reliance” which captures the parties’ attitude of actively seeking help from the court and resorting to litigation to resolve disagreements. This is a quite complex concept that encompasses citizens’ trust in the court as well as their inclination for resolving issues in their own way. From the above discussion, in the context of China, why do people in some provinces rely more on courts to settle disputes, whereas some provinces are indifferent to the court? What influences people’s attitudes toward the court? Unfortunately, previous studies have not provided a direct and clear explanation of this problem, especially in the context of China. In general, the existing literature mainly focuses on judicial transparency, judicial corruption, judicial independence, judicial procedure and judicial professionalism. This paper will measure these aspects by means of a questionnaire.

### Judicial transparency

In 2022, China released the “China Judicial Transparency Index Report (2021),” which believed that judicial transparency mainly includes disclosure of trial information, disclosure of execution information, disclosure of judicial data and disclosure of judicial reform information. With more judicial transparency, will the public be more favorable to the courts? Will citizens turn to transparent courts for help? Previous studies have not reached a consensus on this issue (Meijer, 2009). Literature in political science suggests that transparency in political institutions may hurt citizens’ political trust, because people do not like bargaining and power trading in political institutions (Worthy, 2010; Grimmelikhuijsen et al., 2013; Jenny et al., 2013; Roelofs, 2019). However, compared with other political institutions, judicial institutions have certain particularities. In contrast to government transparency, people respond positively to media exposure of the judiciary and increase the legitimacy of the courts (Gibson and Caldeira, 2009). A recent field experiment study also confirmed this view that judicial transparency indeed positively promotes the public’s trust in judges. The higher the judicial transparency, the more positive the public’s evaluation of the judicial institution (Grimmelikhuijsen and Klijn, 2015). Although judicial transparency in China is relatively low (Feng, 2009), judicial reform in China in recent years has also paid more attention to judicial transparency. In 2013, the Third Plenary Session of the 18th CPC Central Committee called for the improvement of the judicial power operation mechanism and the promotion of open trials. In 2014, the Fourth Plenary Session of the 18th CPC Central Committee also pointed out that we should build an open, dynamic, transparent, and convenient judicial mechanism and promote judicial openness. A particularly key reform took place in 2013 when the SPC promulgated the “Provisions of the Supreme People’s Court on the Issuance of Judgments on the Internet by the People’s Courts” (关于人民法院在互联网剬布裁判文书的规定). Since then, all effective judgments of courts across the country should be published in “China Judgements Online.” Ahl and Sprick (2018) argued that this reform is a sign that Chinese courts are moving toward transparency and the full disclosure of judicial documents will affect the relationship between courts and citizens. In short, China’s judicial transparency has been improving in recent years but unfortunately, there is still a lack of empirical research on the relationship between judicial transparency and public attitudes in China.

### Judicial corruption

Many political studies have shown a certain correlation between government corruption and citizens’ political trust. When citizens perceive the government to be increasingly corrupt, they are less confident in the government’s ability to solve problems and their enthusiasm for political participation is greatly reduced (Seligson, 2002; Anderson and Tverdova, 2003; Goodsell, 2006). As a special political institution, the court also faces the institutional problem of corruption. Li (2012) argued that judicial corruption in China is not a simple summation of individual corruption incidents of judges but has deeper institutional reasons. A study by Gong (2004) also drew a similar conclusion, arguing that judicial corruption is a systemic disease in China. Wang and Liu (2021) used the spatial theory of institutional proximity to explain institutional causes of judicial corruption in China and found that the proximity between political or social institutions causes judicial corruption. The lack of funds is also an important cause of judicial corruption in China. The lack of funds makes it easier for judges to engage in profitable activities and be more vulnerable to external intervention (He X., 2008; Wang, 2013). Previous studies have generally found that the reasons for judicial corruption in China are multifaceted, and a few studies have proved a correlation between judicial corruption and citizens’ trust. Hu (2015) hypothesized that judicial corruption in typical cases usually reduces citizens’ evaluation of judicial departments. He found through a survey that more than half of Chinese respondents “do not trust” judicial departments. Among the reasons given by these respondents, judicial injustice and corruption were the important. In addition, he verified through quantitative analysis that judicial corruption would indeed aggravate citizens’ distrust of the court, resulting in reluctance to use the court as the first choice to solve problems when confronted with disputes (Hu, 2008). Some studies also believe that when citizens realize that the court is a corrupt institution, they will bribe to influence the judgment result to get the desired judgment (Cheeseman and Peiffer, 2022).

### Judicial independence

Judicial independence is the basic symbol of a modern state ruled by law and the main content of constitutional government (Larkins, 1996; Ferejohn, 1998; Carrubba et al., 2008). For a long time, it has been widely believed that the judicial system in China is subject to more interference and less independence (Michelson, 2007; Ng and He, 2017). Although China’s judicial system reform has been deeply constrained by multiple constraints, the realization of judicial independence has always been a goal pursued by the CPC in the construction of the rule of law. After years of judicial system reform, China’s judicial independence is also progressing gradually (Peerenboom, 2008). China’s current constitution and the Organic Law of the People’s Courts both emphasize that “the people’s courts shall exercise judicial power independently in accordance with the law and shall not be subject to interference by administrative organs, public organizations or individuals.” The third and fourth plenary sessions of the 18th CPC Central Committee also emphasized the importance of judicial independence. In the Fifth Five-Year Reform Outline for the People’s Courts (2019–2023; 人民法院第五个五年改革纲要) issued in 2019, the SPC also put forward reform measures to ensure the independence of judge trials. It has created a “pretrial safeguard mechanism,” “process safeguard mechanism,” and “referee safeguard mechanism.” Research has pointed out that the reform of the judicial system of China in recent years has highlighted the “presiding judge responsibility system” as a target; the SPC has changed the past “strong supervision” institutional system to give judges’ discretionary space; judicial reform is also trying to achieve the dual purpose of safeguarding the quality of judicial independence and trials; and the judicial effectiveness and judicial credibility of the people’s courts are constantly improving (Chen, 2015; Fan, 2018; Jiang and Lai, 2020). In recent years, with the increasing independence and authority of the courts, the public has increasingly relied on courts to resolve disputes. At the beginning of China’s reform and opening up (1978), the number of civil cases was 308,000. In 1989, it reached 2.511 million, and in 2019, it has exceeded 10 million (31.567 million). This is a very significant change, which means that citizens are more and more willing to go to court to resolve disputes. So what accounts for this change, and does it mean that the more independent the judiciary, the more citizens will turn to the courts as the primary means of resolving disputes? Especially in the context of China, is there a connection between judicial independence and citizens’ judicial reliance? This requires a further empirical test.

### Judicial procedure

It is generally believed that the fairness of the proceedings is related to the protection of citizens’ procedural rights and affects citizens’ evaluation of the court (Lind and Tyler, 1988; Tyler, 1988). Many studies have shown that litigants’ satisfaction with specific judicial actions will affect their trust in the judicial institution. Whether procedural justice exists is the primary factor for the litigants to voluntarily comply with rules and decisions, and even procedural justice has a greater impact on social attitudes than substantive justice in most cases (Pruitt et al., 1990; Sunshine and Tyler, 2003; Creutzfeldt and Bradford, 2016; Tyler, 2021). Paternoster (1997) argued that litigants who feel that enforcement actions are more in line with procedural justice are more likely to recognize the authority of the law and willing to obey the law. In addition, procedural justice can counteract the negative dissatisfaction caused by a loss, so that the litigant is willing to accept the court’s decision (Jonathan-Zamir et al., 2016). Under the influence of traditional Chinese legal culture, China has been in the habit of attaching importance to substantive justice while ignoring procedural justice (Fu and Cullen, 2008). But this does not mean that procedural justice in China is not important. Especially in recent years, Xi Jinping stressed the need to “let the people in each judicial case feel fair justice,” The SPC began to push hard for “trial-centered” litigation reform in a way that began to learn from the American litigation system. While ensuring substantive justice, more attention should be paid to the legality of the proceedings. In particular, the government has introduced more detailed regulations on litigating rights (Jiang, 2019), rules of evidence (Du, 2020), lawyers’ right to defence (Chen and Kang, 2016), legal aid (Zuo and Zhang, 2019), retrial procedures (Yin, 2020), and others. Some scholars pointed out that due process has “emerged in the morning” in China (He, 2009). Will these efforts win the trust and affirmation of the court from the public? A few relevant empirical studies have focused on China. An empirical study by Su (2014) also showed that if the litigants perceive the proceedings to be fair, they will have higher trust in the courts and are more willing to resort to justice to resolve disputes. Will citizens be more inclined to rely on the courts to resolve disputes because of the fairness of the proceedings? This issue is further discussed in this article.

### Judicial professionalism

The form of modern justice is the product of rationalism in the 19th century. Judicature is a technical, logical, and rational activity that requires judges to be highly professional and elite (Shapiro, 2013). Judicial professionalism in China was systematically rebuilt after the establishment of the People’s Republic of China in 1949. For a long time, the legal quality of Chinese judges has been seriously inadequate. In many places, there have appeared “lazy judges” who only handle a few cases a year, “stupid judges” who lack legal knowledge and skills, and “bad judges” who take bribes and pervert the law (Ye, 2015). Even many veterans became judges, whose legal knowledge and experience was too lacking to handle the complex work of the trial. These problems made citizens less willing to seek the help of the court when they encounter disputes, instead turning to methods such as petition, mediation, and private settlement (He, 1998, He W., 2008; Long, 2008). Therefore, judicial reforms carried out by the SPC since the 1990s are aimed at making judges improve in the direction of “regularization, specialization, and professionalism.” The Judges Law, revised in 2001, requires new judges to pass a national judicial examination. By 2018, more than 210,000 of the 300,000 judicial and legal personnel in the courts had passed the examination and served as judges. The overall quality of judges in China has been improving. Since 2014, the SPC has promoted the reform of the personnel quota system, requiring comprehensive consideration of such factors as judicial performance, professional competence, theoretical competence, and legal work experience to ensure that outstanding judges remain on the front line of trials. In response, local courts have promoted the unified selection, assessment, and appointment of judges. They have also improved the performance assessment requirements for judges, vigorously promoted a judicial accountability system, and constantly improved the treatment of judges (Long, 2018; Wu, 2019). In 2017, China completed a reform of the personnel quota system for judges.^2^ Many scholars indicated that the reform of the personnel quota system made the Chinese judiciary team more elite and “makes judges more like judges,” which is an important transformation of professional judges in China (Li, 2017; Chen, 2018; Gu, 2020). Some literature has shown that improvement of the overall quality of Chinese judges not only involves the optimization of the internal structure of the court, but also brings good social effects, improving the legitimacy of the court. The public will have more trust in the judgment of the court, and citizens will be more willing to seek the help of the court when they encounter disputes (Cao, 2013; Xu and Chen, 2014). This article examines whether that is the case.

This discussion shows that judicial transparency, corruption, independence, procedure, and professionalism all affect and restrict citizens’ recognition and dependence on the court. These factors also reflect why some citizens are more willing to rely on the court to solve problems, whereas others are not. However, existing studies had some shortcomings.

**Figure 2:**
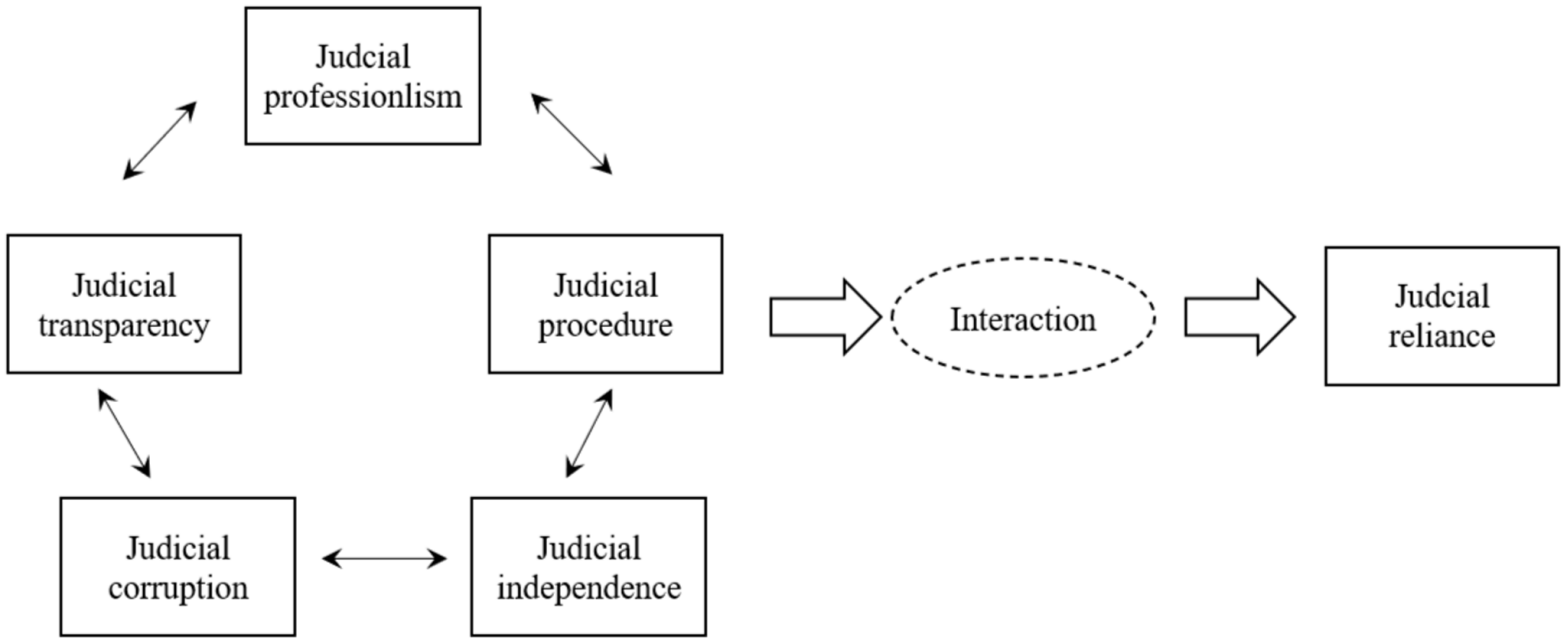
Theoretic framework: judicial conditions and judicial reliance.

First, much of the previous literature theoretically discussed the influence of these five factors on the judicial reliance of citizens, but there have been few concrete empirical discussions, especially in the context of China. Second, most previous studies have been conducted with political trust and public trust as explanatory variables, but public trust is slightly different than the judicial reliance discussed in this article. The research on public trust believes that the court, like other political institutions, also has the anxiety of legitimacy, and the degree of citizens’ approval of the court is particularly important for the development of the court (Brody, 2008), whereas judicial reliance as discussed in this article focuses on citizens’ willingness to resort to the court when they encounter disputes. Third, the previous research mainly involved regression analysis based on linear thinking, but regression analysis is unable to explore the interactive relationships among multiple variables. This article systematically integrates these five judicial variables to explore their influence on citizens’ judicial reliance. Fourth, most prior studies focused on the impact of a single factor on citizens’ judicial reliance. As we know, the causes of the results are often multifaceted. This article systematically integrates the five conditional variables and discusses their interaction with and influence on judicial reliance. Fifth, the discussion of causal mechanisms in previous studies is not enough. Based on the idea of set theory, this article identifies the configuration mechanism that affects judicial reliance based on exploring the covariation trend among different causal conditions. Based on these limitations, this article makes use of fsQCA; integrates and empirically tests the linkage effect of judicial transparency, corruption, independence, procedure, and professionalism on judicial reliance; and deduces the reasons and mechanisms that influence differences in citizens’ judicial reliance in different regions of China. Figure 2 reflects the theoretical framework of this article.

## Data and analytic approach

Our research data are mainly from the PCJI. The report, led by the China University of Political Science and Law, was jointly released by Chinese universities, 38 Chinese judicial organs and legal industry organizations, and 16 international collaboration agencies. The report is the largest, most comprehensive, and authoritative survey and analysis report on the judicial field in China. The purpose of PCJI is to measure the actual situation of judicial operation in China and provide a quantitative assessment tool for the current state of China’s judicial environment.

PCJI conducted a large-scale questionnaire survey in 31 provinces in China, and the respondents mainly included legal professionals (judges, prosecutors, police, lawyers) and the general public. The survey distributed 800 questionnaires in each of 31 provinces in China, including 600 public questionnaires and 200 professional questionnaires. Finally, the total number of samples collected in 31 provinces was 24,012, with a response rate of 97.2%. This survey mainly adopts stratified sampling method, considering different demographic characteristics such as age, education background, occupation, gender and so on, basically guaranteeing the representative sample. All the variables we care about are covered in this survey. The outcome of this article is judicial reliance, which is mainly measured by the question in the PCJI questionnaire: “In your area, how likely is it for the parties to take the initiative to go to court to resolve a dispute?” Causal conditions are judicial transparency, corruption, independence, procedure, and professionalism. The measurement methods are all from the PCJI questionnaire items. Table 1 shows the specific content of the causal conditions and outcome in the questionnaire. PCJI research group assigned scores to each item according to the results of the questionnaire, so the value range of each variable was between 0 and 100.^3^

**Table 1:** Outcome, causal conditions, and measurement.

	Measurement	Respondents
*Outcome*		
Judicial reliance	In your area, how likely is it for the parties to take the initiative to go to court to resolve a dispute?	Judge and Public
*Causal conditions*		
Judicial transparency	1. How likely is it that the court will allow the public to sit in on a trial in your area?	Judge and Public
	2. How likely is it that the court will publish its verdicts promptly according to the law in your area?	Judge and Public
	3. How likely is it that the reasons for the admission and exclusion of evidence are fully explained in the judgment in your area?	Judge
Judicial corruption	1. How likely are judges to have “Guanxi cases” in your area?^a^	Judge
	2. How likely are judges to accept bribes in your area?	Judge and Public
Judicial independence	1. How likely is it that judges will have their decisions interfered with by the leadership of the court in your area?	Judge
	2. How likely is it that judges will have their decisions interfered with by the party and government agencies in your area?	Judge
Judicial procedure	1. How likely is it that the police require the criminal suspect to be self-incriminated in the investigation inquiry in your area?	Judge
	2. How likely is it that attorneys’ right to defend will be protected in your area?	Judge
	3. How likely is it that defendants will receive free legal aid if they cannot afford lawyers in your area?	Public
	4. How likely is it that lawyers will be prosecuted for “lawyer perjury” in your area?	Judge
	5. How likely are lawyers to be insulted by police, prosecutors, or judges in your area?	Judge
	6. How likely is it that the judge will call witness to testify if the defendant asks the witness to testify in your area?	Judge
	7. How likely is it that the court will initiate retrial proceedings to correct a valid judgment that is wrong in your area?	Judge
Judicial professionalism	1. What is the total number of hours of business training you received in the past 3 years?	Judge
	2. How likely are attorneys to make false promises in your area?	Judge and Public
	3. How likely is it that a lawyer will engage in improper dealings with a judge in your area?	Judge and Public
	4. How satisfied are you with your career advancement prospects?	Judge
	5. How satisfied are you with your professional treatment (salary, bonus, welfare, etc.)?	Judge
	6. How satisfied are you with the protection mechanism for fulfilling the responsibilities of the court?	Judge
	7. How much pressure do you feel from performance appraisal?	Judge
	8. How much work pressure do you feel from litigants and your family?	Judge

a “Guanxi cases” is a Chinese expression that refers to a case in which a judge intentionally favors the interests of one party because of personal or human relations during the trial.

In addition to the PCJI, our research also uses first-hand field research data. Since 2016, we have investigated more than 40 courts in Beijing, Shanghai, Shandong, Hubei, Inner Mongolia, and other provinces and conducted in-depth interviews with more than 50 judges. The subjects we interviewed included judges at all levels of the primary, intermediate, and high courts, as well as judges in various chambers of civil, criminal, and administrative trials. Our interviews ranged from one-on-one semistructured interviews to panel discussions with judges. We have accumulated more than 300,000 words of interview transcripts, as well as many first-hand internal court materials, which supplement the data analysis results of this article.

This article mainly uses fsQCA, a set-theoretic configurational approach that is case-based and regards the permutation and combination of causal conditions as configurations that produce an outcome, instead of entities that are examined in isolation (Ragin, 2008; Schneider and Wagemann, 2012). fsQCA was especially popular in politics and sociology in its early years and has gradually expanded to management and education in recent years. This article uses fsQCA (rather than mainstream regression analysis), mainly considering the following factors: First, the sample space is finite. In this article, 31 provinces in China are mainly taken as analysis units, which belong to small and medium-size sample designs in social science analysis. It cannot support the statistical requirements of traditional regression analysis, and it is difficult to use the case study method to analyze sample objects one by one. fsQCA combines the advantages of qualitative and quantitative methods and is especially suitable for research designs with small and medium-size samples. Second, the problem consciousness in this study has causal complexity. This article pays attention to the effect of multiple combinations of conditions on the results. As we have mentioned, we find that whether citizens in a region are willing to resort to the court to solve the problem is not caused by a single reason, but by the combined effects of a variety of conditions. Therefore, this article pays more attention to the identification of multiple conjunctural causation mechanisms than the influence of a single factor. Although we can also achieve this goal by using statistical analysis techniques such as cluster analysis, factor analysis, and item reflection theory, fsQCA has more advantages in this aspect (Rihoux and Ragin, 2008). Third, there may be problems of multi-collinearity among variables in this article. If regression analysis is adopted, the linkage between variables will lead to a larger final standard deviation and wider confidence interval. However, fsQCA focuses more on aggregate effect, so it can effectively avoid the problems of multicollinearity (Ragin, 2008; Du and Jia, 2017).

## Results

### Calibrations for set membership

Before any set analysis, the original data of outcome and causal condition should be converted into set membership with values between 0 and 1 through calibration (Ragin, 2008). Calibration can be divided into direct and indirect methods (Pappas and Woodside, 2021). The direct method requires three qualitative anchors—full in (membership = 0.95), crossover (membership = 0.50), and full out (membership = 0.05)—and then involves calibration with software programs (Fiss, 2011). The indirect method is to recalibrate the original data results to the preset membership distribution based on qualitative evaluation (Cepiku et al., 2020). Different calibration methods should be used for different variable types. In this study, our data are used as continuous numerical variables based on a questionnaire survey, and the direct calibration method is more appropriate (Fiss, 2011).

Because the measurement of each variable in this study is a large-scale questionnaire and there is no interference from extreme values in the data, this study refers to Du and Kim (2021) and selects the maximum and minimum observed values of each variable as the full in and full out of the set, respectively. Referring to Fainshmidt et al. (2019), we use the mean as the crossover. After calculation, we find that the mean and median (50%) are not much different, but using the mean as the crossover could avoid cases being given an accurate membership value of 0.50 and thus, being discarded in subsequent analysis (Fiss, 2011; see Table 2).

**Table 2:** Calibration of causal conditions and outcome.

	Sets	Fuzzy-set calibration		
		Full in	Crossover	Full out
**Outcome**	Judicial reliance	72.6	67.94839	60.9
	Judicial transparency	80.6	75.93871	73.0
**Causal condition**	Judicial corruption	72.9	66.45806	59.8
	Judicial independence	70.4	62.64516	56.2
	Judicial procedure	72.2	68.70000	65.3
	Judicial professionalism	70.1	66.53226	63.1

After the calibration of causal conditions and the outcome, we need to conduct a necessity analysis of causal conditions one by one to identify which causal conditions are necessary to produce the outcome. We then perform a fuzzy-set analysis of the condition to identify the configurations that produce the outcome.

### Necessary conditions analysis

The necessary conditions analysis could identify which causal conditions (including their nonsets) are necessary to produce the outcome. If a causal condition constitutes a necessary condition for the result, it means that once the outcome has occurred, the causal condition must have occurred. In a set relation, the outcome is a subset of its necessary conditions. People usually judge the strength of subset relationships by consistency. The range of consistency is between 0 and 1. The larger the value, the stronger the subset relationship. Existing studies usually use 0.90 as the consistency threshold for the judgment of necessary conditions (Schneider and Wagemann, 2012; Jacqueminet and Durand, 2020).

Referring to previous studies, we analyzed the necessary conditions for the existence of outcome (high judicial reliance) and the nonset of the outcome (low judicial reliance). The results are shown in Table 3, and the consistency of all conditions is less than the threshold standard of 0.90, indicating that no condition can be used as a necessary condition for high (or low) judicial reliance.

**Table 3:** Analysis of necessary conditions for high and low judicial reliance.

Sets of conditions	Judicial reliance		~ Judicial reliance	
	Consistency	Coverage	Consistency	Coverage
Judicial transparency	0.672518	0.785714	0.640884	0.656294
~Judicial transparency	0.705811	0.691578	0.790746	0.679122
Judicial corruption	0.725787	0.768590	0.678867	0.630128
~Judicial corruption	0.650726	0.698052	0.750691	0.705844
Judicial independence	0.659201	0.734818	0.689917	0.674089
~Judicial independence	0.707627	0.722497	0.728591	0.652040
Judicial procedure	0.665860	0.726552	0.629144	0.601717
~Judicial procedure	0.634988	0.661412	0.714088	0.651955
Judicial professionalism	0.715496	0.761108	0.605663	0.564714
~Judicial professionalism	0.590799	0.630899	0.743784	0.696186

~means the absence of; for example: ~ judicial transparency = absence of high judicial transparency.

### Fuzzy-set analysis

Configuration of conditions is used to determine whether and to what extent a set of conditions between multiple conditions constitutes a subset of the outcome. The truth table is constructed based on these causal conditions and outcome, and the threshold is selected according to the recommendations of existing studies and the distribution of the truth table in our analysis. This study is a small-sample analysis, and the minimum case frequency is selected as 1 to retain all truth table rows that may cover any case (Douglas et al., 2020). Second, the consistency threshold is set to 0.80 as recommended by previous studies to ensure sufficient subset relationships (Fiss, 2011). Third, to avoid simultaneous subset relation, we exclude truth table rows with PRI consistency <0.70 (Du and Kim, 2021). Finally, no directional assumptions are made in the counterfactual analysis (Jacqueminet and Durand, 2020), and the default “present or absent” is selected for all conditions, because there is no clear necessary condition and theoretical knowledge guidance.

Based on the asymmetric understanding of configuration causality, we also analyze the nonset of the outcome, i.e., causal configurations with low judicial reliance. However, using the same threshold criteria as the outcome (judicial reliance), the PRI consistency scores for all truth tables remain well below the acceptable 0.70 level and therefore, cannot produce any configuration that meets the criteria. This suggests that although many combinations may lead to low judicial reliance, no qualified configuration emerges when simultaneous subset relationships are excluded. This is similar to observations by Fiss (2011).

Through standardized analysis of filtered truth table rows, three solutions—complex, intermediate, and parsimonious—are obtained. This article adopts the result presentation form proposed by Ragin and Fiss (2008), takes the intermediate configuration as the main reporting object, combines the parsimonious configuration as the reference for determining the core conditions, and finally arranges the configuration analysis results, as shown in Table 4.

**Table 4:** Results of fuzzy analysis.

	Outcome: Judicial reliance					
	C1	C2	C3	C4	C5	C6
Judicial transparency	⊗	⊗		●	●	●
Judicial corruption	●	●	●	⊗		⊗
Judicial independence		●	●	⊗	⊗	
Judicial procedure	⊗		⊗		●	●
Judicial professionalism	●	●	●	●	●	●
Raw coverage	0.384	0.417	0.361	0.385	0.383	0.378
Unique coverage	0.021	0.022	0.015	0.033	0.031	0.000
Consistency	0.959	0.943	0.946	0.923	0.959	0.927
Overall solution coverage	0.587					
Overall solution consistency	0.905					

● = core condition present; ⊗ = core condition absent; ● = peripheral condition present; ⊗ = peripheral condition absent.

Configuration 1 (C1) indicates that citizens in a region will trust the courts more if the level of judicial professionalism is high, even though judicial corruption is relatively serious, judicial transparency is relatively low, and judicial procedure is not so fair. The public still more likely to turn to the courts for help in disputes. Among them, judicial corruption and judicial professionalism are the core conditions, whereas judicial procedure and judicial transparency are the peripheral conditions. Of the 38.4% cases that can be explained to produce high judicial reliance, 2.1% can be explained by this configuration.

Configuration 2 (C2) shows that although judicial corruption is relatively serious and judicial transparency is relatively low, a region with a high degree of judicial independence and professionalism will also have a very high degree of judicial reliance. Among them, judicial corruption and judicial professionalism are the core conditions, whereas the other conditions are peripheral conditions.

Configuration 3 (C3) shows that even if judicial corruption is serious and judicial procedure fairness is low in a region, a high level of judicial professionalism and independence will still lead to a high degree of judicial reliance in this region. Judicial corruption and judicial professionalism are the core conditions, and the other conditions are peripheral conditions.

Configuration 4 (C4) indicates high judicial transparency and a high level of judicial professionalism as the core conditions, and a low level of judicial corruption and judicial independence as the peripheral conditions, will also bring about higher judicial reliance in a region.

Configuration 5 (C5) indicates that a high level of judicial professionalism and transparency as the core conditions, complemented by a high level of judicial procedure fairness and low level of judicial independence, will also lead to a high degree of judicial reliance in a region.

Configuration 6 (C6) indicates that if a region has a low level of judicial corruption, a high level of judicial professionalism, a relatively fair judicial process, and a high level of judicial transparency, under the interaction of these conditions, the region will also have a high degree of judicial reliance.

In conclusion, C1–C6 describe possible reasons for high judicial reliance in different regions of China. Interestingly, judicial professionalism has always been an indispensable core condition in these six configurations. The configurations can also be further classified to generate two large types of judicial ecology: The first type is “high corruption + high judicial professionalism” represented by C1–C3. Although judicial corruption is not a good thing, judicial professionalism seems to offset the negative effects brought by judicial corruption and will eventually bring high judicial reliance to a region. The second type is “high judicial transparency + high judicial professionalism” represented by C4–C6, which indicates that if a region has high judicial transparency and a high level of judicial professionalism of local judges, combined with other conditions, a region will also have high judicial reliance.

### Robustness checks

According to Wu et al. (2021), this article adjusts multiple analysis indicators to cope with the threat of fsQCA parameter setting. The results of robustness tests are shown in Table 5. The baseline refers to the baseline model reported in this article. On this basis, we first up-adjust the original consistency and PRI cutoff value of truth table analysis to 0.95 and 0.76, respectively, and the number of configurations are reduced to five; C2 does not appear. Second, we adjust the calibration anchor to 90%, 50%, and 10% of the data distribution (Campbell et al., 2016), and 75%, 50%, and 25% (Park and Mithas, 2020). The original consistency and PRI consistency thresholds are appropriately adjusted according to the distribution of the truth table, and the number of configurations is further reduced. But the configurations form the subset of the base model. In conclusion, after the change in sensitive parameters, except for the change in configuration number, there is no change in each configurations component or only subset change, indicating that this result is robust (Wu et al., 2021).

**Table 5:** Results of robustness checks.

Model	Calibration anchors	Threshold	Intermediate solutions	Subset
Baseline	Max, Mean, Min	0.92/0.72	~transparency*corruption* ~ procedure*professionalism	C1
			~transparency*corruption*independence*professionalism	C2
			corruption*independence* ~ procedure*professionalism	C3
			transparency* ~ corruption* ~ independence*professionalism	C4
			transparency* ~ independence*procedure*professionalism	C5
			transparency* ~ corruption*procedure*professionalism	C6
Increase in consistency	Max, Mean, Min	0.95/0.76	~transparency*corruption* ~ procedure*professionalism	C1
			~transparency*corruption*independence*professionalism	C2
			corruption*independence* ~ procedure*professionalism	C3
			transparency* ~ corruption*procedure*professionalism	C4
			transparency* ~ independence*procedure*professionalism	C5
Changing calibration anchors	90th, 50th, 10th	0.81/0.65	~transparency*corruption* ~ independence* ~ procedure*professionalism	C1 subset
			transparency*corruption*independence* ~ procedure*professionalism	C3 subset
	75th, 50th, 25th	0.84/0.75	~transparency*corruption* ~ independence* ~ procedure*professionalism	C1 subset
			transparency*corruption*independence* ~ procedure*professionalism	C3 subset

## Discussion: China and judicial professionalism

When citizens encounter disputes, will they actively seek the help of the court? It is generally believed that the position of Chinese courts in the political system is relatively weak, and the courts are often weak in regulating social relations and resolving social conflicts. In this context, it is also a conditional strategic consideration for citizens to seek the help of the courts. So, under what circumstances will Chinese citizens rely more on the courts to settle disputes? What is the logic and mechanism behind this? This is the main issue of this article. We use judicial reliance to describe the willingness of citizens to resort to judicial means when they encounter disputes. Our research finds obvious local differences in the judicial reliance of citizens in different provinces of China. So, we use fsQCA to explore the legal dependence of 31 provinces in China, and identify five causal conditions that may affect the judicial reliance of China according to the existing theories—namely, judicial transparency, corruption, independence, procedure, and professionalism—to discuss what judicial conditions will create a region with higher judicial reliance.

Based on the perspective of configuration analysis, we find that the existence of any of these five causal conditions alone was not sufficient to lead to high judicial reliance, which reminds us that we should pay more attention to the interaction and joint effects among these conditions. In our study, we identify two mechanisms that lead to high judicial reliance: The first mechanism is “high judicial corruption + judicial professionalism” (C1–C3), wherein although corruption in the judicial system is not a good thing, other conditions in the coordination of the judicial system, combined with judicial professionalism as an offsetting effect, still can bring higher judicial reliance to a region. This mechanism also suggests that if judicial corruption is relatively serious in a region, litigants are more likely to bribe the court to reach a favorable decision. The second mechanism is “high judicial transparency + high judicial professionalism” (C4–C6). If a region has high judicial transparency, “visible justice” can help citizens trust in the decisions of the court, and with the interaction of other conditions, citizens’ judicial reliance will also be enhanced. These two mechanisms have a common feature: judicial professionalism is the core condition throughout. Among the six configurations identified by fsQCA, each configuration has judicial professionalism as the core condition. This shows that judicial professionalism is very important to citizens’ judicial reliance and even the benign operation of the judicial system. To a certain extent, our research conclusions complement those of previous studies, which generally only focus on the influence of a single factor on judicial reliance, but fail to analyze how multiple factors interweave and affect judicial reliance. For example, as mentioned above, existing studies only confirmed the influence of judicial corruption on judicial reliance, but did not verify how judicial corruption and other related factors interact and jointly affect judicial reliance (Hu, 2015). Our study supplemented the literature in this field to a certain extent, and also inspired the academic community to pay attention to the influence of interaction between different factors on judicial reliance. To some extent, our research conclusions complement those of previous studies, which generally only focus on the impact of a single factor on judicial reliance, but fail to analyze how multiple factors interwoven to affect judicial reliance.

Why is professionalism so important to Chinese courts? The development of judicial professionalism in China has not formed gradually with a long process of institutional change, as in European and American countries. China’s judicial professionalism efforts were systematically rebuilt after the founding of the People’s Republic of China in 1949. Due to the late start, the quality of judges in local courts was uneven, often resulting in different judgments in the same case. Even some local courts often caused a lot of unjust, false, and wrong cases due to the lack of professional quality of judges. Therefore, the SPC has always taken professionalism as the core direction of judicial reform.

After the CPC established a new political power in 1949, the basic framework of the court was preliminarily constructed. The people’s court is an integral part of the people’s democratic power and the mass line (群众路线), and the relevant institutional system of judicial professionalism is seriously insufficient (He, 2005). After the reform and opening up in 1978, the construction of the rule of law in China began to get on track, and the construction of judicial professionalism began. In 1983, Jiang Hua, then president of the SPC, stressed at a conference on judicial administration that the training of trial judges should be strengthened. Since the 1990s, the construction of judicial professionalism has been fully rolled out in courts at all levels in China. Starting from the revision of the Civil Procedure Law in 1991, China began to try to introduce elements of the British and American defence systems in court trials.

In 1995, China’s first Judges Law was promulgated, systematically regulating the appointment and removal procedures, educational requirements, and access conditions for judges. In 2001, China revised the Judges Law, stipulating that new judges must pass the National Judicial Examination. With the revision and improvement of the Civil Procedure Law, the Criminal Procedure Law and the Administrative Procedure Law, China’s litigation procedures are becoming more standardized and professional. In recent years, Zhou Qiang, president of the SPC, has repeatedly emphasized the importance of professionalism construction, and “normalization, specialization, and professionalism” have always been the direction of court reform. Some recent reforms are also sending a signal of professionalism. For example, the SPC has been vigorously promoting the guiding case system. Guiding cases are judgment cases issued by the SPC with typical supervision and guiding significance and have taken legal effect. Different from the common law jurisprudence, China’s guiding cases only have “reference” significance for judges’ decisions, and are not compulsory binding force. So far, the SPC has issued 28 batches of guiding cases, totalling 162 cases. Ahl (2014) pointed out that the guiding case system can ensure the uniformity of judicial application in multiple jurisdictions, which is a significant sign of the promotion of judicial professionalism by Chinese courts. In addition, in 2019, China promulgated the Opinions on Further Optimizing the Allocation of Judicial Resources and Comprehensively Improving Judicial Efficiency (关于进一步优化司法资源配置全面提升司法效能的意见), which required that “the range of civil and commercial cases to which the sole-judge trial system applies shall be adequately expanded, and the courts at the primary level shall be encouraged to adopt a pattern in which most cases are heard by a sole judge and certain cases are heard by a collegial bench.” This indicates that more and more cases will be heard by judges acting alone (previously collaboratively). Judges can independently competent for trial work, which also shows that the professionalism of Chinese judges has made a certain progress.

Our research also shows that judicial professionalism is also a key factor contributing to citizens’ judicial reliance, and as we have seen, if the professionalism level is higher, local citizens will have more trust in the court and when they encounter a conflict, will be more willing to go to court to solve the problem. When citizens’ judicial reliance is relatively high, it may also explain how judicial professionalism can foster citizens’ perceptions of the rule of law. It may also enhance the legitimacy of the court. So why should the professionalism of judges affect citizens’ willingness to participate? Combined with our previous first-hand field research materials, it can be summarized in two aspects.

One the on hand, the higher the level of judicial professionalism, the stronger the comprehensive quality and professional ability of the judges, ensuring people will generally think that their rulings will be fairer. A judge at a primary court in Western China once told us that there was a very simple civil dispute case. The court had appointed a young judge who had just graduated to try the case, but the litigant challenged the court, hoping to replace the judge with someone who had more trial experience (Interviewee CS). The litigant felt that the young judge was not professional enough and worried that his decision would not be fair (Interviewee AR). As a young judge put it:

As a young judge fresh out of college, I was often viewed with suspicion by litigants who didn’t think I had a lot of trial experience and worried that my decisions would be unfair. Litigants tend to prefer judges who appear to be experienced to hear their cases (Interviewee MK).

One the other hand, the higher the level of professionalism of the judges in the court, the more they will be able to make the same judgment in the same case and make judgments more in line with expectations. This is evident in commercial cases. Lawyers entrusted by companies involved in disputes are very concerned about which judge oversees their cases. If the judge has rich trial experience, they may predict the possible outcome of the judgment based on previous precedence (Interviewee BW). As a judge said:

Commercial cases are generally professional, so judges need to have high professional knowledge. For example, disputes over construction project contracts [建设工程合同纠纷] require the judge to be able to understand the construction contracts. These contracts are very complex and involve a lot of technical terms. So, a case of this level of difficulty obviously requires a judge with more legal skills (Interviewee CG).

In short, we can be relatively optimistic that the construction of judicial professionalism in China will continue. As China’s legal environment becomes better, more citizens will rely more on the institutionalized channels provided by the courts to resolve disputes, and the stability mechanism of Chinese society will be gradually strengthened.

This article leaves a lot of room for further study and discussion. First, the research data in this article are small-sample data. If we want to systematically investigate the judicial reliance of Chinese citizens, we will need to rely on larger survey data. Second, the units of analysis in this article are mainly at the provincial level in China. To further understand local differences in citizens’ judicial reliance in China, the units of analysis need to be lowered to the city level or even the county level. Meanwhile, this article mainly uses the second-hand survey data released by the government. Although this set of data shows the development of China’s judiciary in all aspects, it also has certain limitations. The information richness reflected in this data is not enough, the details of sampling are not clearly explained, and the quality of respondents’ answers has not been systematically verified. Therefore, it is necessary to combine more first-hand data for further analysis in the future. Third, this article only preliminarily confirms the importance of judicial professionalism to citizens’ judicial reliance, but to further understand how judicial professionalism affects citizens’ judicial reliance and what impact it has on judicial system reform, mixed research methods are needed. Fourth, this article only analyses under what conditions citizens will actively choose judicial means to solve problems when they encounter disputes. But the unanswered question is what other options citizens may have if they do not seek the court’s help and what strategic considerations citizens may have in front of those options. This is a very interesting question that needs further study by matching more data on mediation, arbitration, petition, etc. Fifth, the issue of citizens’ attitudes toward judicial institutions is an extremely large and complex research field. This article only touches on a certain aspect of citizens’ political attitudes in the context of China. Does the tendency of Chinese citizens’ attitudes toward judicial institutions conform to the rules found in other countries? How is China’s story different from the world’s story? This is an issue that can be further discussed in future comparative judicial politics studies.

## Data availability statement

The raw data supporting the conclusions of this article will be made available upon request to the corresponding author, without undue reservation.

## Ethics statement

Ethical review and approval was not required for the study on human participants in accordance with the local legislation and institutional requirements. Written informed consent from the patients/participants or patients/participants legal guardian/next of kin was not required to participate in this study in accordance with the national legislation and the institutional requirements.

## Author contributions

XW: conception, design, data analysis, and interpretation. CG and YL: study design. SZ: provision of study materials, conception. CG and YL: collection and assembly of data. XW, CG, YL, and SZ: manuscript writing. All authors contributed to the article and approved the submitted version.

## Funding

This work was supported by the Tianjin Social Science Foundation of China (TJZZ21-008). The funders had no role in the study design, data collection and analyses, the decision to publish, or the preparation of the manuscript. Additionally, the funders had no influence on the interpretation of data and the final conclusions drawn.

## Conflict of interest

The authors declare that the research was conducted in the absence of any commercial or financial relationships that could be construed as a potential conflict of interest.

## Publisher’s note

All claims expressed in this article are solely those of the authors and do not necessarily represent those of their affiliated organizations, or those of the publisher, the editors and the reviewers. Any product that may be evaluated in this article, or claim that may be made by its manufacturer, is not guaranteed or endorsed by the publisher.

## Footnotes

^1^Judicial local protectionism, is mainly refers to in the judicial process, the local administrative organs for the various interests consideration for judicial organs in the process of case acceptance, trial and execution of administrative intervention, the influence of judiciary case acceptance, trial and execution, in order to reach the purpose of protecting local interests, department, And the judicial independence, fairness, justice and efficiency.

^2^The personnel quota system for judges in China mainly refers to the classification of court personnel by means of selection, which can be divided into three categories: trial personnel, trial auxiliary personnel and judicial administrative personnel. Only trial officers are judges within the quota system, while the other two categories are staff outside the quota system. The aim is to streamline the number of judges and improve their professionalism.

^3^For the specific calculation method and process, see Zhang et al. (2020), Report on Chinese Justice index 2019, China University of Political Science and Law Press.
