# Supplementary material for: Why do people choose courts to resolve disputes? A fuzzy-set analysis of Chinese citizens’ judicial reliance
Source: Front Psychol. 2023 Jan 5;13:1015987. doi: 10.3389/fpsyg.2022.1015987 (PMC9849697; doi:10.3389/fpsyg.2022.1015987)
Supplement: Supplementary file 1 [file Data_Sheet_1.docx]

**APPENDIX**


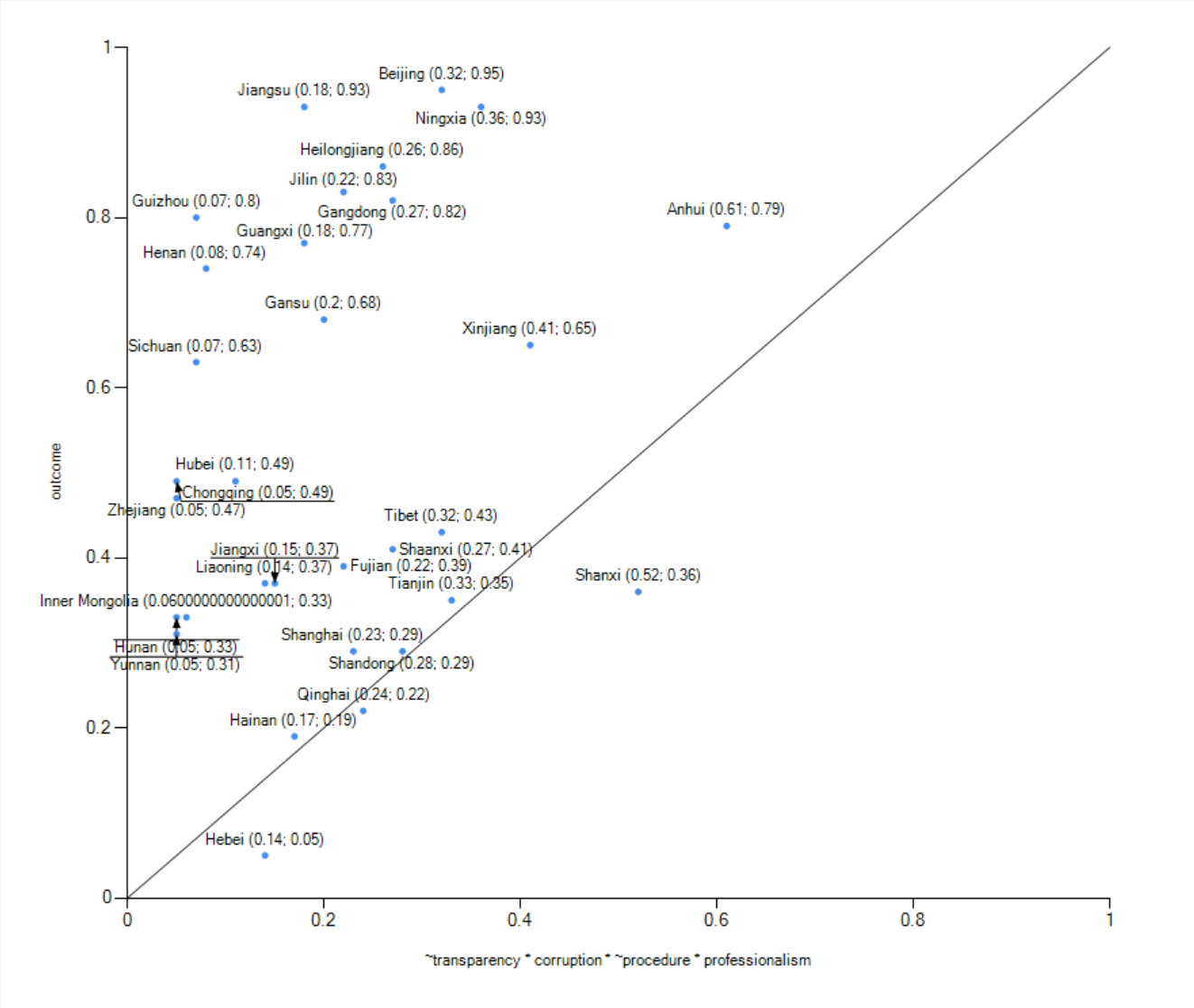


Figure A1. ~transparency*corruption*~procedure*professionalism（C1）


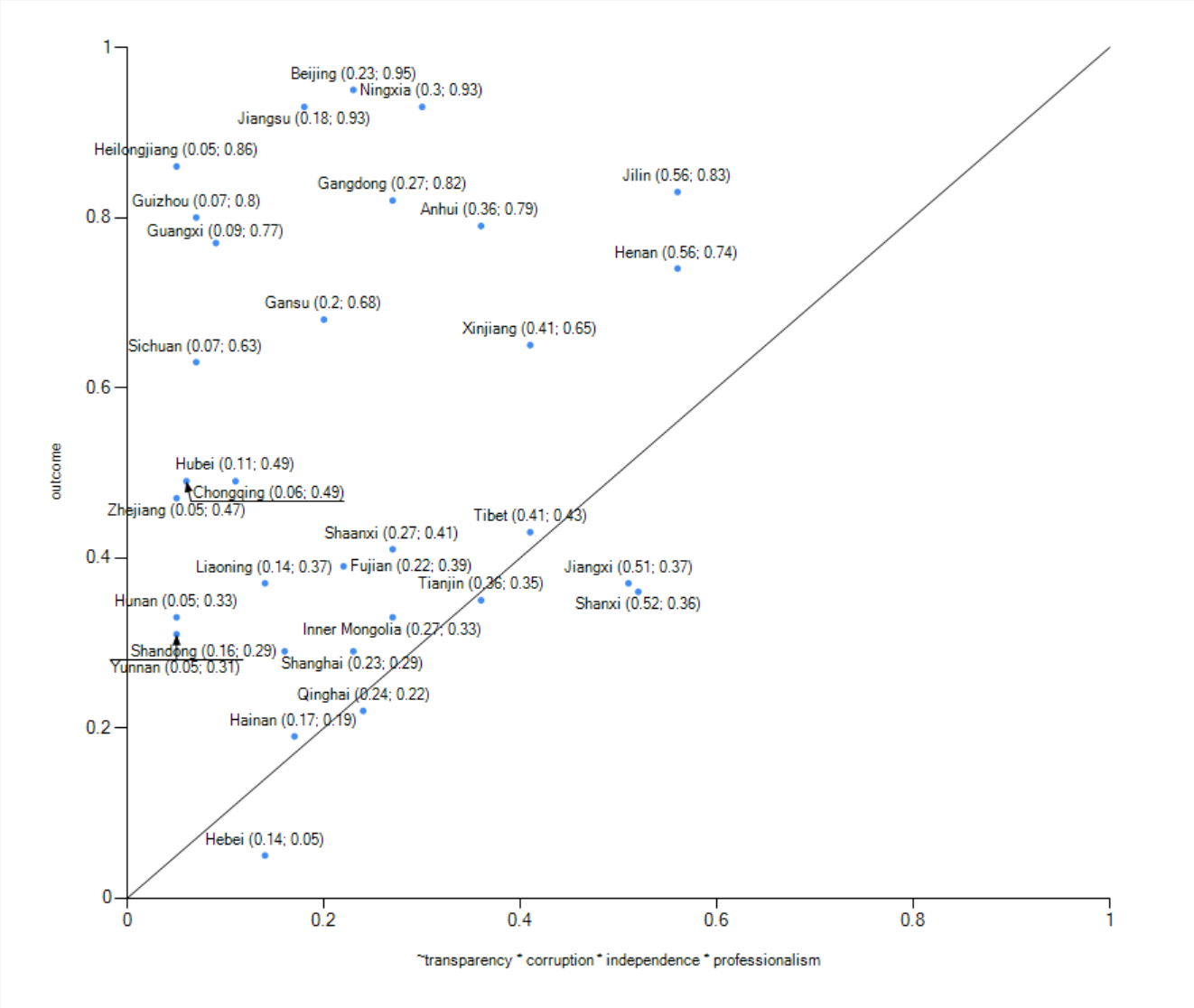


Figure A2. ~transparency*corruption*independence*professionalism（C2）


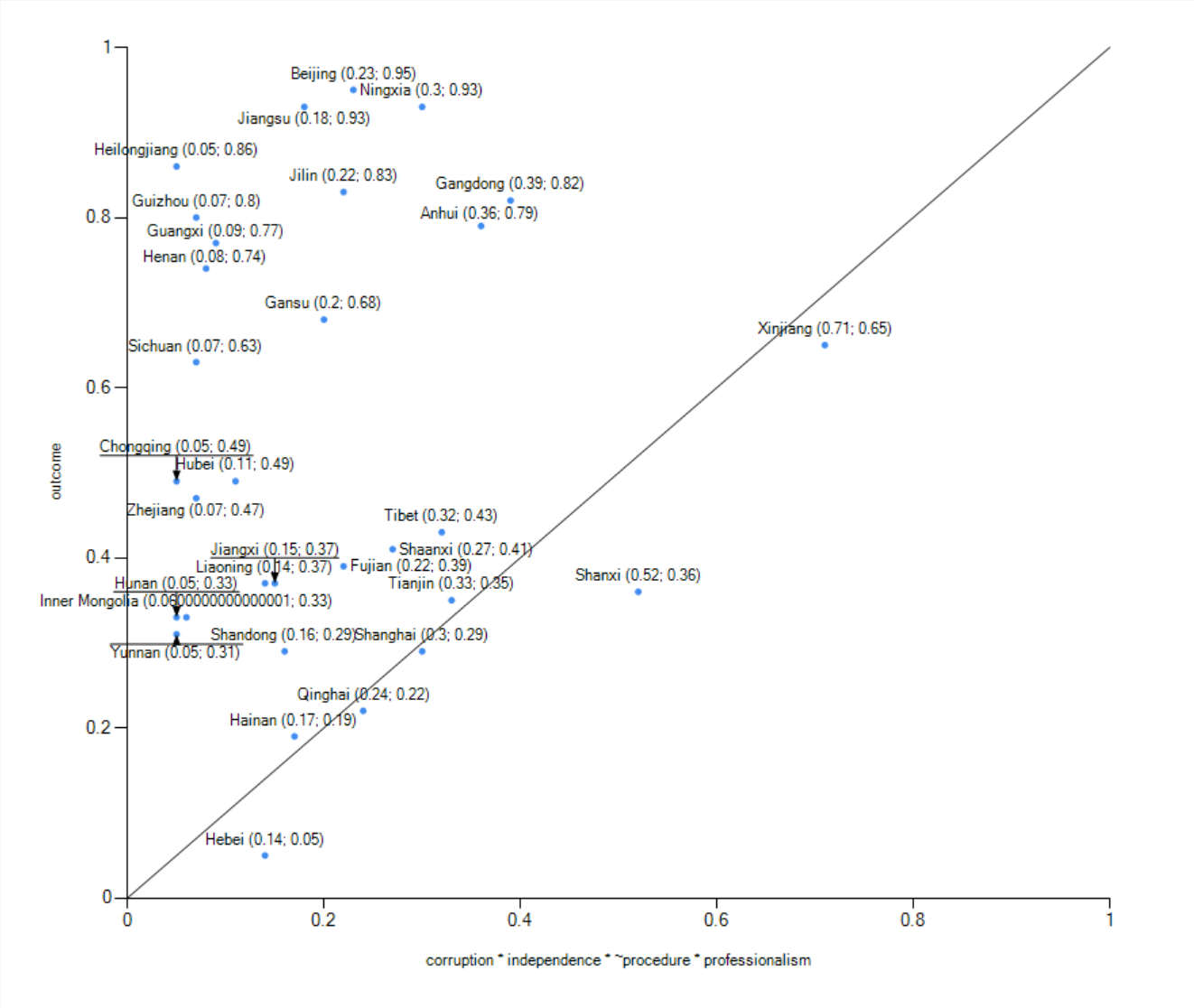


Figure A3. corruption*independence*~procedure*professionalism（C3）


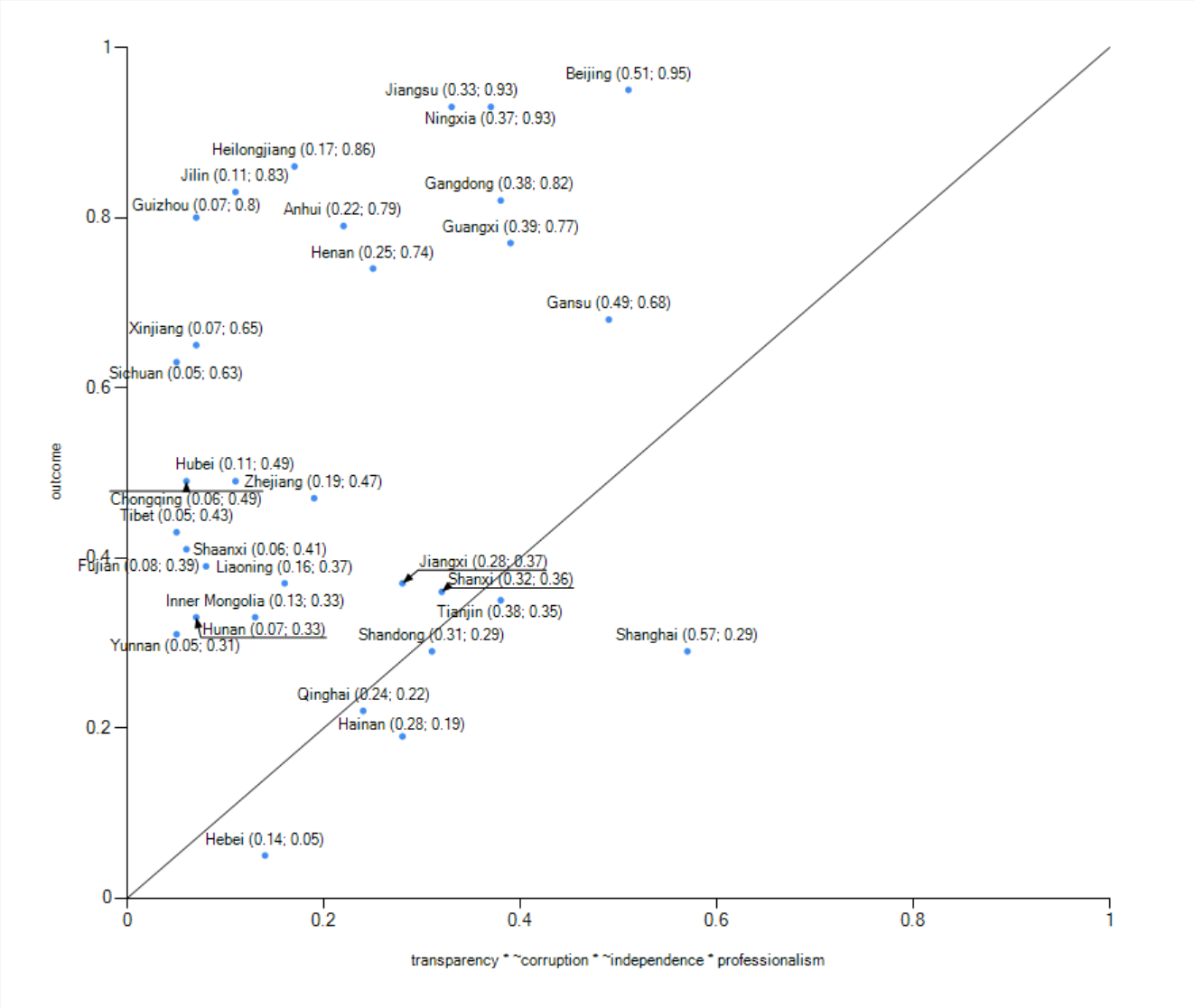


Figure A4. transparency*~corruption*~independence*professionalism(C4)
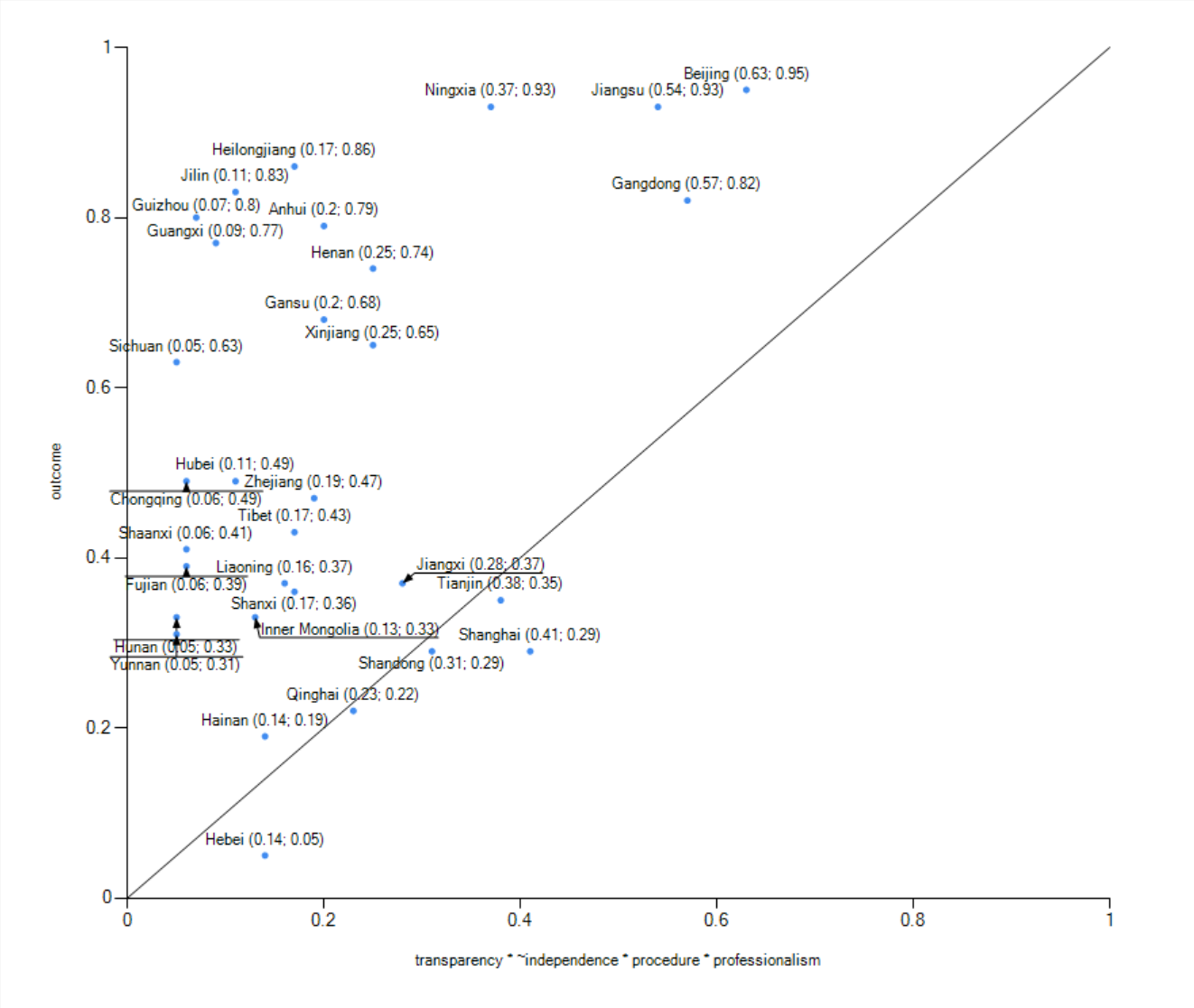


Figure A5. transparency*~independence*procedure*professionalism（C5）


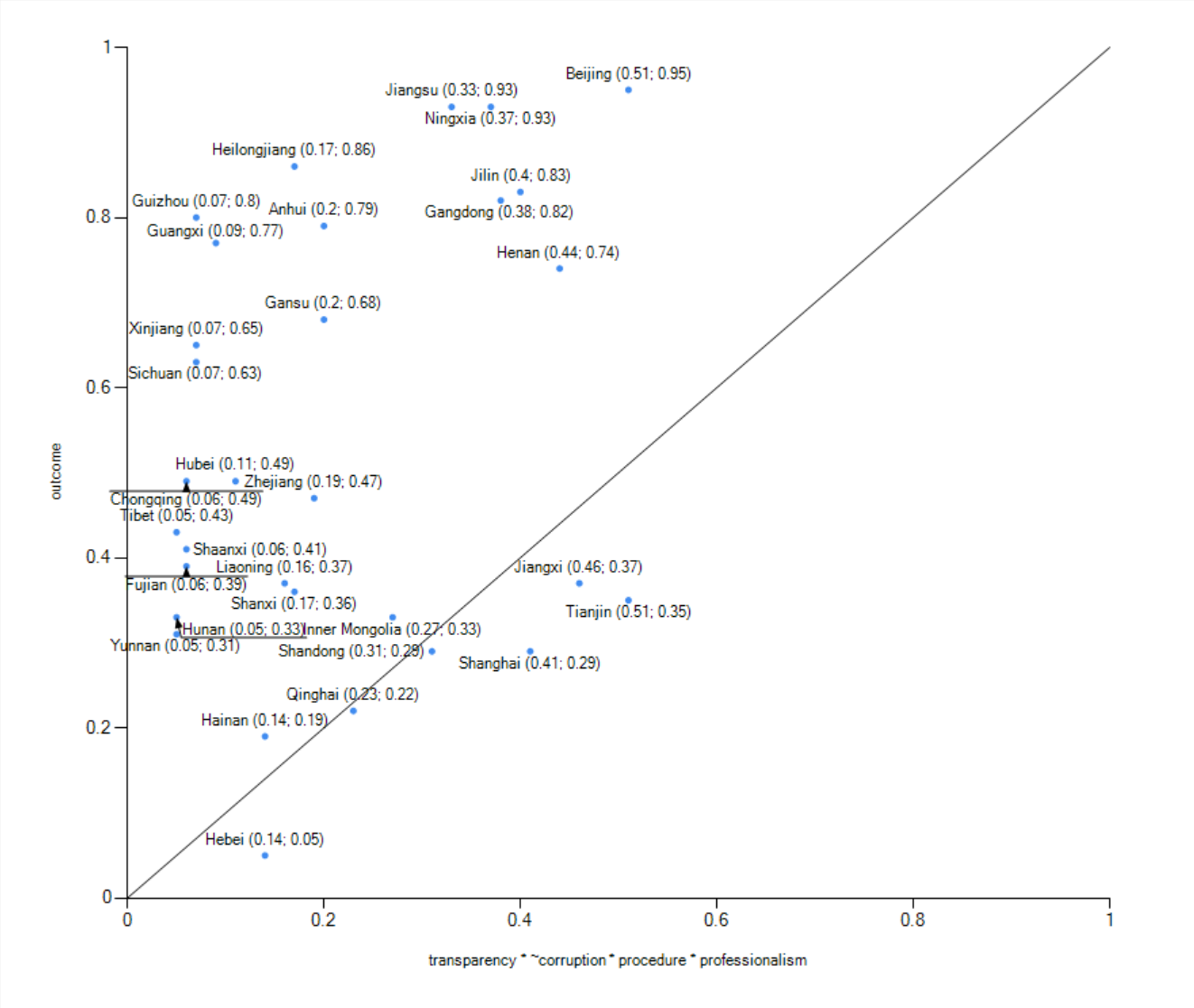


Figure A6. transparency*~corruption*procedure*professionalism（C6）
